# Case report: Severe pneumonia and pneumomediastinum in a previously robust adolescent caused by Omicron BA.5.2

**DOI:** 10.3389/fmed.2023.1132630

**Published:** 2023-04-17

**Authors:** Tianxin Xiang, Jianhua Fang, Tao Cheng, Zhongmin Li, Daxian Wu, Shouhua Zhang, Shanfei Ge, Wei Zhang

**Affiliations:** ^1^Department of Hospital Infection Control, The First Affiliated Hospital of Nanchang University, Nanchang, China; ^2^Jiangxi Hospital of China-Japan Friendship Hospital, Nanchang, China; ^3^Department of Infectious Disease, The First Affiliated Hospital of Nanchang University, Nanchang, China; ^4^Department of Respiratory Medicine, The Second People's Hospital of Shangrao, Shangrao, China; ^5^Department of General Surgery, Jiangxi Provincial Children's Hospital, Nanchang, China; ^6^The Department of Pulmonary and Critical Care Medicine, The First Affiliated Hospital of Nanchang University, Nanchang, China; ^7^Jiangxi Clinical Research Center for Respiratory Diseases, The First Affiliated Hospital of Nanchang University, Nanchang, Jiangxi, China

**Keywords:** COVID-19, Omicron, mediastinal emphysema, severe pneumonia, adolescent

## Abstract

The manifestation of severe pneumonia is only occasional, and pneumomediastinum is a condition that occurs rarely in Coronavirus disease 2019 (COVID-19) patients, especially in those patients who are infected with the Omicron variant. In addition, whether severe pneumonia or pneumomediastinum often occurs in patients in older age, in poor physical condition, or with underlying diseases remains to be ascertained. To date, severe pneumonia and pneumomediastinum due to Omicron infection had not been reported in a young patient with an excellent physical condition. In this study, we report such a case with the aforementioned manifestations in a robust adolescent infected with Omicron BA.5.2.

## Introduction

Coronavirus disease 2019 (COVID-19), which is caused by severe acute respiratory syndrome coronavirus-2 (SARS-CoV-2), remains a challenging disease that is impacting global health and economy, especially when the virus mutates. Omicron is a variant of SARS-CoV-2, which is characterized by a short incubation period, rapid transmission, and a strong immune evasion ability, and it has become the current epidemic strain around the world (1). Compared with the original virus strain, the Omicron virus has a lower ability to replicate in the lung tissue and a weaker ability to cause lung disease. It rarely causes significant alterations in imaging of the lungs (2). Omicron usually causes mild upper respiratory tract infection symptoms, including sore throat, runny nose, and headache (3). The manifestations of severe pneumonia is only occasional and emphysema in the mediastinum is extremely rare. Additionally, pneumonia or pneumomediastinum often occurs in patients in older age, in poor physical condition, or with underlying diseases (4). In this study, we reported the first case of severe pneumonia and pneumomediastinum in a previously robust adolescent infected with Omicron BA.5.2.

## Case presentation

A 16-year-old Chinese student was admitted to the Second People's Hospital of Shangrao, China, with fever for 2 days and was diagnosed with COVID-19 on 10 November 2022. The virus strain was then confirmed to be Omicron BA.5.2 by sequencing. He had no prior illness and had practiced martial arts in Shaolin Temple. He had contact with confirmed SARS-CoV-2 patients 5 days before the onset of illness and lived in the case-reporting community.

**Figure 1:**
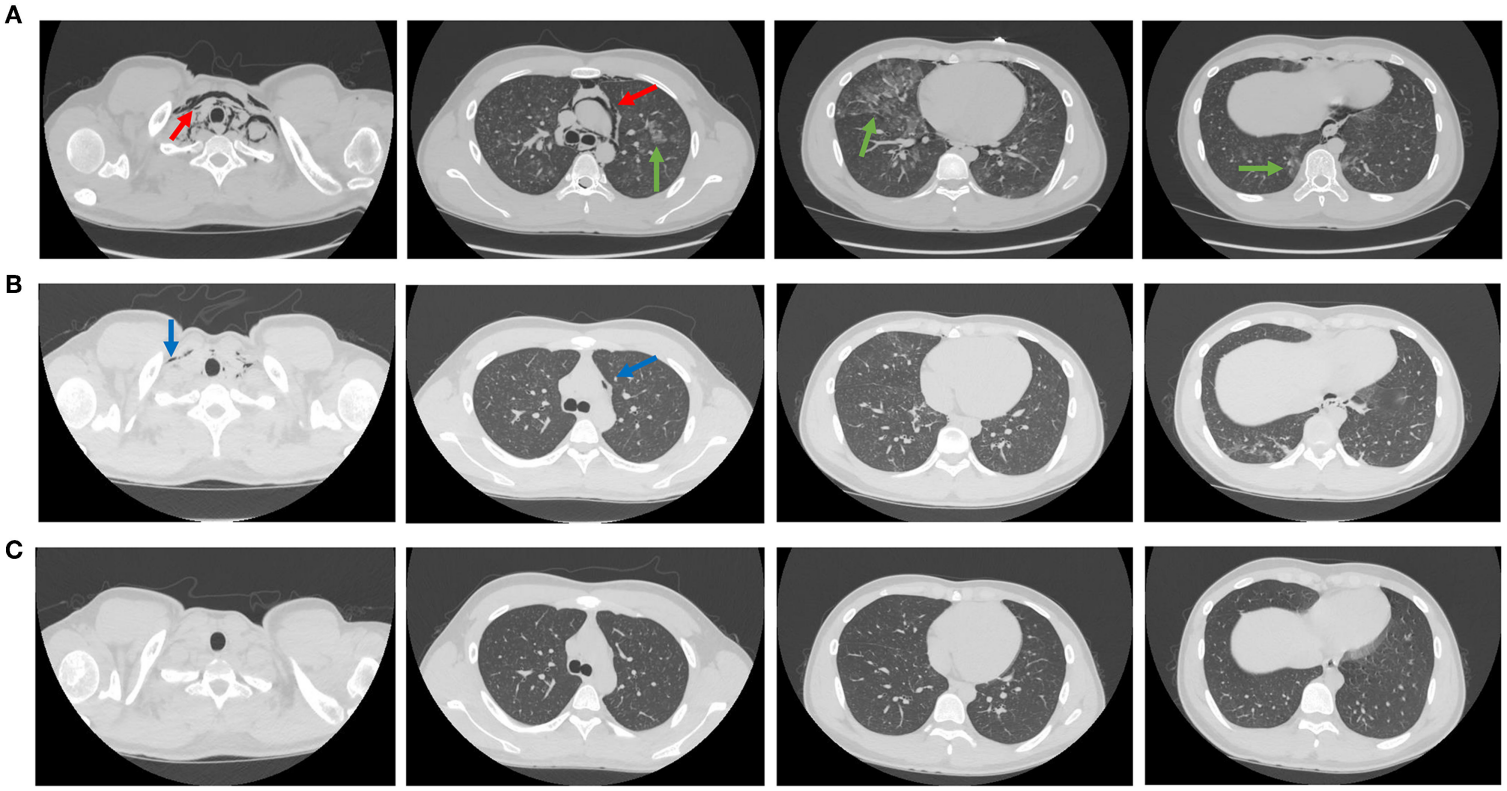
The manifestations of lungs and mediastinum in computed tomography. The CT manifestations of lungs and mediastinum on 12 November 2022. The injuries indicated by the red arrow were pneumomediastinum, which spreads to the base of the neck. The injuries indicated by the green arrow were mottled and patchy ground glass opacities **(A)**. The CT manifestations of lungs and mediastinum on 15 November 2022. The injuries indicated by the blue arrow were pneumomediastinum that has improved significantly **(B)**. The CT manifestations of lungs and mediastinum on 21 November 2022. The pneumomediastinum and ground glass opacities were completely absorbed **(C)**.

At the beginning of the disease course, the main symptom was slight fever. The highest temperature recorded in the patient was 38.5°C, which was accompanied by cough, mainly dry cough, and occasionally purulent expectoration. In addition, symptoms of headache, sore throat, muscle ache, nasal congestion, dry throat, nausea, and vomiting did not manifest. The next day, the fever subsided and the cough improved slightly. However, on 12 November 2022, the patient suddenly experienced an onset of chest tightness, dyspnea, and a slightly aggravated cough. The oxygen saturation decreased to 90% without oxygen inhalation, and the oxygenation index was 250 mmHg or less. The patient was urgently transferred to the First Affiliated Hospital of Nanchang University. Emergency chest computed tomography (1.15 mm per slice) presented bilateral diffuse centrilobular lesions, multiple ground glass opacities with blurred boundaries, and some thickened interlobular septa located in both lobes. In addition, bilateral emphysema at the base of the neck and mediastinum was found, with a few pneumothoraxes on the right side (Figure 1). The respiratory rate was at least 30 breaths per minute and the oxygen index was 250 mmHg or less. Leukocyte and lymphocyte counts were 6.63 × 10^9^/L and 0.33 × 10^9^/L, respectively. The percentage of lymphocytes was 5.0%. The level of C-reactive protein was 18.64 mg/L. The routine tests for Epstein–Barr virus, cytomegalovirus, herpes simplex virus, adenovirus, respiratory syncytial virus, mycoplasma, chlamydia, and influenza viruses A and B were negative. Antibodies related to autoimmune diseases were also undetectable. Cultures of blood, urine, and bone marrow were sterile. After admission, conservative treatments were adopted for the patient. Adequate oxygen was administered immediately *via* inhalation. Nematvir/Ritonavir, human interferon alpha 2b (α – 2b), human immunoglobulin, and ambroxol were also prescribed. The patient recovered smoothly after those treatments, and alterations in the lungs were significantly improved on 15 November 2022 and almost absorbed on 21 November 2022.

## Discussion

The strain involved in this case was the evolutionary branch of the SARS-CoV-2 Omicron variant BA.5.2, which is the variant with the most profound variation and the most mutations in the current epidemic strain (5). Compared with the Wuhan standard strain, the Omicron strain has 78 mutation sites, 108 missing sequences, and 68 blank sequences, indicating that the virus was not significantly related to B.1.1.529 detected in South Africa for the first time in 2021. As reported in previous studies, the Omicron variant contains at least 32 mutations in the spike protein, which is double the number of variants than the Delta variant, which increases infectivity and immune escape of the Omicron variant compared with the early wild-type strain (6, 7). In addition, more than 80% of patients infected with the Alpha strain had viral pneumonia on pulmonary CT images. Nevertheless, only about 1% of patients infected with the Omicron variant had pulmonary foci (8). Hence, as the most highly mutated strain, Omicron's transmission and immune evasion capacities were drastically enhanced compared with other variants. However, pathogenic ability was obviously decreased (9). Therefore, severe pneumonia in Omicron-infected patients is rare, and pneumomediastinum is even rarer. Most cases of pneumomediastinum were reported before the Omicron discovery, and almost all patients were elderly or had combined kinds of underlying diseases (10). In previous studies (11, 12), COVID-19 patients with pneumomediastinum underscored the strong correlation between the severity of COVID-19 and the underlying diseases, such as hypertension, diabetes, asthma, dyslipidemia, kidney disease, acute lymphoblastic leukemia, and pulmonary embolism. The patient, in our case, was a 16-year-old student who had been vaccinated against SARS-CoV-2 even practiced martial arts in Shaolin Temple. He also was found to be in an excellent physical condition. However, he rapidly progressed to developing severe pneumonia and pneumomediastinum after the Omicron BA.5.2 infection.

It is unclear whether this phenomenon was attributed to an increased pathogenicity of Omicron BA.5.2 under certain conditions or whether the patient has a hereditary susceptibility to Omicron BA.5.2, and future studies should investigate these hypotheses. However, the present study reported the first case of severe pneumonia and pneumomediastinum in a previously robust adolescent which was caused by the emerging Omicron BA.5.2, and thus, it is unique for this case.

Pneumomediastinum is classified into spontaneous and secondary pneumomediastinum based on whether there is a specific responsible pathologic cause or not. It is considered to occur spontaneously even when there is no definite etiology, unlike secondary pneumomediastinum, which occurs with an apparent causative factor (13). The traumatic causative factors included chest and abdominal injuries and even iatrogenic operations such as endoscopic procedures, central venous catheterization, and intubation. The non-traumatic diseases included asthma, chronic obstructive pulmonary disease, interstitial lung disease, and malignancy (14, 15). Thus, patients with secondary non-traumatic pneumomediastinum were always immunocompromised, but spontaneous pneumomediastinum occurred in young immunocompetent patients.

Pneumomediastinum is usually caused by an internal rupture or a tear in the esophagus or trachea, which is more common in elderly patients with severe diseases, especially in patients with intubation (16). The exact pathophysiological mechanisms of pneumomediastinum in COVID-19 patients have not been fully elucidated. The mainstream view of previous studies suggested that they may be due to the rapid increments in pressure differences across the alveolar membrane, resulting in terminal alveolar rupture with air leakage to the mediastinum, known as the “Macklin Phenomenon (17).” Another hypothesis that we propose is that some structural and pathological alterations occur in the lung parenchyma of COVID-19 patients. Those alterations included downregulation of the surfactant, loss of extracellular matrix and basement membrane, damaged type 2 pneumocytes, and hypercoagulability (18). Cough and underlying diseases are also mentioned as contributing factors. The patient, in our case, is a robust young man with no underlying comorbidities, and he also does not possess a tall and thin frame (BMI 23.3). In addition, he also did not suffer from frequent cough and tracheal intubation episodes. Hence, we postulated that structural and pathological injuries caused by the virus were the main mechanisms of pneumomediastinum in this case.

Dyspnea is non-specific and a common symptom of severe COVID-19 pneumonia, pneumothorax, and pneumomediastinum. In this case, the patient's condition also deteriorated with chest tightness, dyspnea, and rapid oxygen desaturation, which prompted us to believe that those syndromes could indicate pneumothorax or pneumomediastinum. Once excessive gas accumulates in the mediastinum, the large veins and nerves will be compressed, leading to an accelerated heart rate and dyspnea and even a hypotensive shock (4). It was reported that the mortality rate of spontaneous pneumomediastinum was as high as 28.5% (5). Therefore, clinicians should pay great attention to the aforementioned factor, although the incidence rate is meager. Previous literature reported that the prognosis of pneumomediastinum with previous irreversible lung diseases, such as pulmonary fibrosis and emphysema, was worse than that of pneumomediastinum with unknown etiologies (spontaneous), and the leakage of gas would disappear after 20 days (19). However, the case in this study did not have any underlying diseases, and its emphysema almost disappeared in about 5 days. Therefore, consistent with a previous study, we also endorse that the underlying conditions strongly impact the prognosis of pneumomediastinum in COVID-19 patients (20).

In conclusion, we reported the first case of severe pneumonia and pneumomediastinum in a previously robust adolescent infected with Omicron BA.5.2. This case reminds us that severe complications could still have happened in the pathogenicity-attenuated Omicron variant and even in a person with an excellent physical condition. Timely diagnosis and therapy of severe pneumonia and pneumomediastinum are crucial to its prognosis.

## Data availability statement

The raw data supporting the conclusions of this article will be made available by the authors, without undue reservation.

## Ethics statement

Written informed consent was obtained from the minor(s)' legal guardian/next of kin for the publication of any potentially identifiable images or data included in this article.

## Author contributions

TX and JF drafted the manuscript. JF performed virological analysis and contributed to interpretation. TC and ZL collected the data. DW and SG contributed to the interpretation and also critically revised the manuscript. WZ was incharge of the critical revision of the manuscript. All authors contributed to the article and approved the submitted version.
